# Lifelong TMAO exposure exerts hypotensive effects in aged spontaneously hypertensive rats

**DOI:** 10.3389/fcvm.2025.1645530

**Published:** 2025-08-29

**Authors:** Wojciech Kopacz, Kinga Jaworska, Mateusz Koper, Marcin Ufnal

**Affiliations:** Department of Experimental Physiology and Pathophysiology, Laboratory of the Centre for Preclinical Research, Medical University of Warsaw, Warsaw, Poland

**Keywords:** bacterial metabolites, hypertension, TMAO, TMA, cardiovascular system

## Abstract

Trimethylamine N-oxide (TMAO), a bacterial metabolite associated with cardiovascular risk, has a debated role, with evidence suggesting both harmful and protective effects. We hypothesized that chronic TMAO supplementation modulates hypertension-related complications through alterations in the tissue renin-angiotensin system (RAS). Ten-week-old male spontaneously hypertensive rats (SHRs) were divided into two groups, receiving either water or TMAO-supplemented water for 80 weeks. Circulatory parameters, including hemodynamics, echocardiographic measurements, and pathomorphological assessments, tissue RAS expression and biomarker profiles, were evaluated. Rats treated with TMAO exhibited significantly higher plasma TMAO levels accompanied by significantly lower mean and systolic blood pressure and NT-proBNP levels. In the TMAO group, diuresis and natriuresis were numerically increased, and the left atrial size was smaller. No pathological effects of TMAO on the circulatory system were observed in rats. Echocardiographic parameters and age-related histopathological changes, including fibrosis in the heart and kidneys, were comparable between groups. TMAO-treated rats showed upregulated expression of RAS components, including angiotensinogen and AT1b and AT2 receptors, in the heart, kidneys, and colon. TMAO supplementation also increased blood and urinary levels of trimethylamine, a toxic precursor and metabolite of TMAO. In conclusion, lifelong exposure to TMAO improves circulatory function in hypertension, as evidenced by reduced blood pressure, lower NT-proBNP levels, and decreased left atrial size—findings consistent with a reduction in cardiac workload. The upregulation of RAS components may represent a compensatory response to the blood pressure-lowering effect of TMAO. However, the concurrent rise in potentially toxic TMA levels warrants further investigation to fully assess the long-term safety of TMAO supplementation.

## 1 Introduction

Among various mechanisms by which microbiota influence health, bacterial metabolites have gained attention as potential diagnostic or prognostic biomarkers and therapeutic targets. One such metabolite, trimethylamine N-oxide (TMAO), has emerged as a focal point due to its association with cardiovascular risk (1). Numerous studies have investigated TMAO's efficacy as a biomarker, with the strongest evidence supporting its prognostic value in various cardiovascular diseases (2).

TMAO is formed through the hepatic oxidation of trimethylamine (TMA), a metabolite derived from bacterial processing of choline, carnitine, and related compounds, which are abundant in red meat (3). Initially, this led to the hypothesis that TMAO might link red meat consumption with increased cardiovascular risk. However, TMAO is also present in dietary sources like fish and seafood (4), which are generally considered heart-healthy, complicating its interpretation as a risk factor.

This paradox has driven substantial research aimed at clarifying TMAO's role in cardiovascular pathology. Current evidence remains inconclusive, with debate surrounding whether TMAO acts as a harmful agent, part of an adaptive physiological response, or merely a marker without direct involvement in pathophysiological processes (5, 6). For instance, TMAO has been implicated in the progression of heart failure (7–9) and the development of atherosclerosis (10), potentially by promoting platelet hyperreactivity and increasing thrombosis risk (11). Conversely, other studies have found no association between TMAO and atherosclerosis development (12), and some even suggest that TMAO may exert protective effects on the heart (13) and against atherosclerotic lesions (14).

Although cardiovascular diseases are predominantly associated with aging populations, most studies are conducted on young animal models. Spontaneously Hypertensive Rats (SHR) begin to exhibit increased mortality from cardiovascular complications at approximately 15 months of age, with mortality rates reaching up to 80% by 21 months (15).

The duration of the present study enables a comprehensive evaluation of the lifelong impact of TMAO while minimizing potential data loss associated with high mortality rates.

This study is one of the very few studies that performed a long-term assessment, concluding with aged rats. Notably, no studies to date have examined the effects of lifelong TMAO exposure on circulatory homeostasis, leaving critical gaps in our understanding of its long-term impact.

The present study evaluated the effect of lifelong TMAO exposure on survival and cardiovascular pathology. Specifically, we hypothesized that TMAO exerts a protective effect against cardiovascular complications associated with hypertension by modulating the tissue renin-angiotensin system (RAS). To test this hypothesis, we supplemented hypertensive rats with TMAO for 80 weeks and evaluated circulatory system parameters as well as the expression of RAS components across various tissues.

## 2 Materials and methods

### 2.1 Animals

The study was performed according to the guidelines from Directive 2010/63 EU of the European Parliament on the protection of animals used for scientific purposes and approved by the 2nd Local Bioethical Committee in Warsaw (permission: WAW2/046/2022). Male Spontaneously Hypertensive Rats (SHR) were obtained from the Central Laboratory for Experimental Animals, Medical University of Warsaw (Warsaw, Poland). Animals were housed (three to four per cage), in polypropylene cages with environmental enrichment, under a daily cycle of 12 h light and 12 h darkness, temperature 22–23°C, humidity 45%–55%, fed standard laboratory diet (Labofeed B standard, Kcynia, Poland) and water *ad libitum*. All animals were carefully monitored throughout the study by trained personnel and under the supervision of a licensed veterinarian. Animals were checked daily for general health and well-being. If any signs of significant distress, pain, or suffering were observed, whether related to the natural course of disease, aging, or any other cause, humane endpoints were applied, and the animals were euthanized to prevent unnecessary suffering. The following clinical signs were used as criteria for humane endpoints and were closely monitored throughout the study: rapid or severe weight loss (>20% of baseline body weight), persistent or severe lethargy, lack of grooming, labored breathing or respiratory distress, inability to eat or drink, severe dehydration, neurological signs (e.g., seizures, loss of coordination), signs of severe infection (e.g., abscesses, open wounds, discharge), tumor burden interfering with normal function, persistent hunched posture or reluctance to move, and signs of severe pain unresponsive to analgesia. Animals exhibiting one or more of the above signs were humanely euthanized in accordance with approved protocols, using methods consistent with AVMA Guidelines for the Euthanasia of Animals.

### 2.2 Experimental protocol

10-wk-old rats were maintained for 2 days in metabolism cages to evaluate 24 h water and food balance. Afterwards, rats were randomly assigned to two groups: SHRs drinking tap water (SHR-Water group, *n* = 10) or SHRs drinking TMAO water solution (333 mg/L, Sigma-Aldrich, St. Louis, MO, USA; SHR-TMAO group, *n* = 10) for 80 weeks, which corresponds virtually to life-long exposure. The dose of TMAO was selected based on our previous studies (13, 16) to increase the plasma TMAO concentration by 3–5 times to mimic possible physiological concentrations.

After 80 weeks of supplementation, survived rats from the SHR-Water (*n* = 8), and SHR-TMAO (*n* = 8) groups were maintained immediately for 2 days in metabolism cages to evaluate 24 h water and food balance and to collect urine for biochemical analysis. Next, rats underwent echocardiography using a Samsung HM70 ultrasound system equipped with a linear probe at 5–13 mHz. The probe was placed on the chest wall to obtain images from the right parasternal short axis. The next day, rats were anesthetized with urethane (1.5 g/kg bw ip, Sigma-Aldrich) and the left femoral artery was cannulated for ABP and HR recordings as described above for 20-wk-old rats. Blood samples were collected and animals were sacrificed by exsanguination. Heart, kidney, liver, jejunum, and colon tissues were collected, and subjected to molecular biology and biochemistry studies as described below.

### 2.3 Serum and urine TMAO, TMA, and general biochemistry evaluation

Serum and urine TMAO and TMA were evaluated using a Waters Acquity Ultra Performance Liquid Chromatograph coupled with a Waters TQ-S triple-quadrupole mass spectrometer, as previously described (13). In brief, samples were prepared using the derivatization technique. The mass spectrometer was operated in multiple-reaction monitoring (MRM)-positive electrospray ionization (ESI+) mode for all analytes. Analyte concentrations (TMA and TMAO) were calculated using a calibration curve prepared by spiking water with working stock solutions. Serum and urine samples were compared against the calibration curve.

Plasma and urine Na+, creatinine, and urea were analyzed using Cobas 6,000 analyzer (Roche Diagnostics, Indianapolis, IN).

Glomerular filtration was estimated by creatinine clearance, based on serum and urine creatinine levels, and calculated with the formula: ClCr = urine creatinine (mg/dl) × urine flow (ml/min)/serum creatinine (mg/dl).

### 2.4 NT-proBNP

ELISA kit was used for the evaluation of NH2-terminal pro-B-type natriuretic peptide (NT-proBNP; FineTest Biotech Inc., USA). All procedures were performed according to the standard protocol of ELISA kit operating instructions. The absorbance intensity was measured at 450 nm with the Multiskan microplate reader (ThermoFisher Scientific, Waltham, MA). All experiments were performed in duplicates.

### 2.5 Quantitative RT-PCR for the tissue expression of renin-angiotensin system

The expression of renin, angiotensinogen (AGT), angiotensin-converting enzyme 2 (ACE2), angiotensin receptor type 1a (AT1a receptor), angiotensin receptor type 1b (AT1b receptor), and angiotensin receptor type 2 (AT2 receptor) expression in the heart, kidney cortex, kidney medulla, liver, jejunum and colon tissue samples.

Approximately 25 mg of the tissue was homogenized in lysis buffer (Qiagen, Hilden, Germany) in a TissueLyser bead mixer (Qiagen) at 25 Hz in two 5 min repetitions. Total cellular RNA was extracted with Trizol reagent (Invitrogen, Carlsbad, CA, USA) according to the manufacturer's protocols. RNA was quantified by measuring absorbance at a wavelength of 260 nm using a spectrophotometer NanoDrop 1000. Next, using an Iscript® (Bio-Rad, Hercules, CA, USA), 1.5 µg of the total DNAse-treated RNA was reverse-transcribed. Real-time quantitative PCR analysis was performed via a Bio-Rad real-time system using gene-specific primer pairs (Bio-Rad, Hercules, CA, USA). The amplified products were detected with iTaq® Universal SYBR Green Supermix (Bio-rad, Hercules, CA, USA). Melting curve analysis and agarose gel electrophoresis of the PCR products confirmed amplification specificity. Data analysis was performed using Bio-Rad CFX Maestro Software (version 0.953, MOMA, Aarhus, Denmark). Transcript levels were normalized relative to the reference gene GAPDH. All reactions were run in duplicate.

### 2.6 Histopathological evaluation of the heart and kidneys

Heart and kidney tissue sections fixed in 10% buffered formalin were processed through graded ethanol and xylene baths for dehydration and embedded in paraffin wax. Thin sections (3–4 μm) were stained with hematoxylin and eosin for general histology and van Gieson stain to visualize connective tissue fibers. Histopathological evaluations were conducted at magnifications of ×10, ×40, and ×100 (objective lens) with a ×10 eyepiece, and photographic documentation was captured.

The kidneys were examined for glomerulosclerosis, thickening of glomerular capillary basal membranes, lymphocytic and histiocytic infiltration of the interstitium, calcification, fibrosis, basophilia and cystic lesions of convoluted tubules in renal cortex, degeneration of the renal arteries (hypertrophy, vacuolization, hyalinization of the vascular smooth muscle, arterial inflammation, necrosis and fibrosis). The heart was examined for left ventricular free wall hypertrophy, hypertrophy of cardiomyocytes, cytoplasm vacuolization (cardiomyocytes), perivascular fibrosis in myocardium, increased infiltration of mononuclear cells in myocardium, subpericardium and subendocardium. These parameters were evaluated semi-quantitatively, using a scale of 0–4, with 0 meaning no pathological changes observed, and 4 meaning severe morphological changes. Summary histopathological injury scores for heart and kidney were calculated. Fibrosis levels were quantified as the percentage of connective tissue relative to the total surface area. Micrographs were taken at ×40 (objective lens) with a ×10 eyepiece using an Olympus BX41 light microscope and CellSens software (Olympus, Tokyo, Japan). For each sample, 10 fields of the myocardium or kidneys were analyzed, and fibrosis percentages were calculated with ImageJ software (National Institutes of Health, Bethesda, MD) as previously described (13).

### 2.7 Data analysis and statistics

In the acute hemodynamic experiments, mean arterial blood pressure (MABP), diastolic blood pressure (DBP), systolic blood pressure (SBP), and heart rate (HR) were calculated on the blood pressure tracing by AcqKnowledge 4.3.1 Biopac software (Biopac Systems). Cardiac output calculated as stroke volume × HR. Total peripheral resistance calculated as MABP/cardiac output.

The Shapiro–Wilk test was used to test normality of the distribution. Accordingly, differences between the groups were evaluated by *t*-test or Mann–Whitney. A value of two-sided *P* < 0.05 was considered significant. Analyses were conducted using Dell Statistica (version 13, Dell, Tulsa, OK).

## 3 Results

### 3.1 Survival, body mass, food intake, and water-electrolyte balance

Two rats from each group died before the end of 80-week observation period. Specifically, from the SHR-Water group one rat was euthanized due to dyspnea (post-mortem lung edema), and one died spontaneously (post-mortem lung edema), at the age of 85, and 86 weeks, respectively. Two rats from SHR-TMAO group were euthanized due to the reasons unrelated to cardiovascular complications, namely cachexia (post-mortem neoplasma), and armpit abscess, both at the age of 75 weeks.

In survived rats, there were no significant differences between the SHR-Water and SHR-TMAO groups in body mass, food intake, diuresis and water intake (Table 1). However, rats on TMAO tended to have increased urine output (*p* = 0.08).

**Table 1:** Metabolic and circulatory parameters in hypertensive rats maintained on water (SHR-water, *n* = 8) or TMAO solution (SHR-TMAO, *n* = 8).

Group/Parameter	SHR-Water	SHR-TMAO	*p*
Survival, energy and water balance			
Survival from the study onset (%, n)	80% (8/10)	80% (8/10)	-
Body mass (g)	384.71 ± 16.51	375.24 ± 16.35	0.27
24 h food intake (g)	23.91 ± 3.89	19.06 ± 6.28	0.09
24 h stool output (g)	12.86 ± 3.35	10.55 ± 2.93	0.16
24 h water intake (g/100 g of b.w.)	11.72 ± 1.93	12.98 ± 2.27	0.32
24 h urine output (g/100 g of b.w.)	7.19 ± 1.87	9.06 ± 1.84	0.08
TMA/TMAO balance			
Serum TMAO (µmol/L)	1.63 ± 0.7	23.6 ± 9.41	0.001
Serum TMA (µmol/L)	<LOQ	0.2 ± 0.16	-
Urine TMAO (µmol/L)	262.5 ± 134.50	7,120.5 ± 3,734.09	0.005
Urine TMA (µmol/L)	0.98 ± 0.23	9.67 ± 5.05	0.003
Biochemical parameters in serum			
NT-proBNP (pg/ml)	61.2 ± 13.75	37.4 ± 13.13	0.04
Serum urea (md/dl)	72.5 ± 21.68	84.0 ± 8.25	0.27
Serum creatinine (mg/dl)	0.49 ± 0.23	0.51 ± 0.13	0.84
Serum glucose (mg/dl)	257.5 ± 99.38	271.3 ± 14.98	0.94
Serum sodium (mmol/L)	117.17 ± 4.45	118.5 ± 6.41	0.69
Biochemical parameters in urine			
Glucose in urine (mg/dl)	31 ± 24.4	25.5 ± 27.93	0.72
Urine sodium (mmol/L)	19.66 ± 6.53	30.88 ± 12.48	0.12
24 h sodium urine excretion (mmoles)	0.46 ± 0.15	0.73 ± 0.3	0.13
Urine protein	198.4 ± 44.59	215.3 ± 46.64	0.54
Urine creatinine	38.3 ± 11.83	51.8 ± 30.64	0.35
Creatinine clearance (ml/min)	1.55 ± 0.86	1.92 ± 1.47	0.62
Hemodynamic parameters			
Systolic blood pressure (mmHg)	130.71 ± 13.23	104.48 ± 17.95	0.01
Diastolic blood pressure (mmHg)	79.18 ± 20.79	59.92 ± 11.20	0.08
Mean blood pressure (mmHg)	102.56 ± 17.14	81.22 ± 13.31	0.03
HR (beats/min)	294.59 ± 60.31	258.55 ± 47.16	0.27
Cardiac output (ml/min)	80.6 ± 43.88	84.31 ± 30.23	0.59
Total peripheral resistance (mmHg*min/ml)	1.69 ± 0.47	1.10 ± 0.45	0.09
Echocardiographic parameters			
IVSs (cm)	0.42 ± 0.07	0.37 ± 0.05	0.42
IVSd (cm)	0.27 ± 0.05	0.31 ± 0.05	0.11
LVDd (cm)	0.51 ± 0.09	0.53 ± 0.18	0.86
FS (%)	33.75 ± 6.61	32.00 ± 9.32	0.67
EF (%)	68.25 ± 7.94	64.5 ± 11.87	0.47
LA (cm)	0.57 ± 0.07	0.53 ± 0.07	0.11
PWD (cm)	0.36 ± 0.05	0.33 ± 0.07	0.32
LVDs (cm)	0.34 ± 0.07	0.36 ± 0.14	0.54
PWS (cm)	0.44 ± 0.04	0.43 ± 0.07	0.54
Ao (cm)	0.48 ± 0.04	0.44 ± 0.07	0.23
LA/Ao	1.2 ± 0.16	1.18 ± 0.11	0.76
LVEDV (ml)	0.34 ± 0.15	0.43 ± 0.32	0.5
LVESV (ml)	0.11 ± 0.06	0.16 ± 0.14	0.38
SV (ml)	0.25 ± 0.08	0.33 ± 0.15	0.32
Organ mass			
Heart mass (g)	2.04 ± 0.26	1.87 ± 0.12	0.17
Kidneys mass (g)	2.61 ± 0.19	2.54 ± 0.17	0.46

Creatinine clearance calculated as urine creatinine × urine output (ml/min)/plasma creatinine. Cardiac output calculated as stroke volume × heart rate. Total peripheral resistance calculated as mean arterial blood pressure/cardiac output. IVSd, intraventricular septum in diastole (cm); IVSs, intraventricular septum in systole (cm); LVDd, ventricular diameter in diastole (cm); FS, fractional shortening (%); EF, ejection fraction (%); LA,- left atrium (cm); PWD, posterior wall diastolic diameter (cm); LVDs, left ventricular diameter in systole (cm); PWS, posterior wall systolic diameter (cm); Ao, aorta (cm); LA/Ao, left atrium/aorta; LVEDV, end-diastolic volume of the left ventricle (ml); LVESV, end-systolic volume of the left ventricle (ml), Values are means, ±SD. *P* values by *t*-test or Mann–Whitney *U* test.

Similarly, there were no differences with respect to biochemical plasma and urine parameters, as well as creatinine clearance (Table 1), but SHR-TMAO group showed numerical increase in sodium urine concentration and 24 h Na+ urine excretion in comparison to SHR-Water group.

### 3.2 TMA/TMAO balance

SHR-TMAO group had higher TMAO serum concentration and higher TMAO urine excretion than SHR-Water group. Also, SHR-TMAO exhibited higher TMA urine excretion. TMA concentrations in serum in SHR-Water group were below the limit of quantification (Table 1).

### 3.3 Circulatory parameters

The SHR-Water and SHR-TMAO group showed hypertrophic cardiomyopathy with compromised systolic functions (Table 1). Rats maintained on TMAO had lower mean and systolic blood pressure and showed a tendency towards lower diastolic blood pressure and lower total peripheral resistance (*p* = 0.03, *p* = 0.01, *p* = 0.08, and *p* = 0.11 respectively; Table 1) than SHRs on water. Also, SHR-TMAO group had decreased plasma NT-proBNP (*p* = 0.04) and tended to have lower left atrial size and heart mass than the SHR-Water group (*p* = 0.16) (Table 1).

There were no significant differences between the groups with respect to basic echocardiographic parameters (Table 1).

### 3.4 Histopathology of the heart and kidney

The SHR-Water and SHR-TMAO group showed signs of hypertrophic cardiomyopathy. In cardiac myocytes, slight signs of hypertrophy and decreased cell striation were observed. The histopathological picture was dominated by myocardial fibrosis and inflammatory cell infiltrates. Fibrosis involved mainly the wall of the left ventricle and was concentrated around the coronary vessels and in the papillary muscles, sometimes also subendocardially. The inflammatory infiltrate was of a mixed nature, with lymphocytes dominating with foci of histiocyte and neutrophil infiltrates. The described cellular composition indicates an active process of myocardial damage causing cardiac myocyte disintegration.

In kidneys, vascular changes included arteries of all sizes from small arterioles to large arteries. The histopathological picture of blood vessels was dominated by hypertrophy of smooth muscle cells. Moreover, degenerative changes in the form of vascularization and hyalinization of the arterial muscles were also visible. Changes were also found in the renal corpuscles in the form of renal corpuscle degeneration - glomerulosclerosis and thickening of basement membranes. Interstitial changes were observed in the renal cortex as interstitial fibrosis processes and infiltration of mononuclear cells—lymphocytes and macrophages. Renal parenchyma fibrosis spread particularly around small and medium-sized arteries of the renal cortex and also involved severely damaged renal corpuscles. The presence of tubular cysts has been observed in the renal tubules, which may be due to chronic progressive nephropathy (CPN).

**Figure 1:**
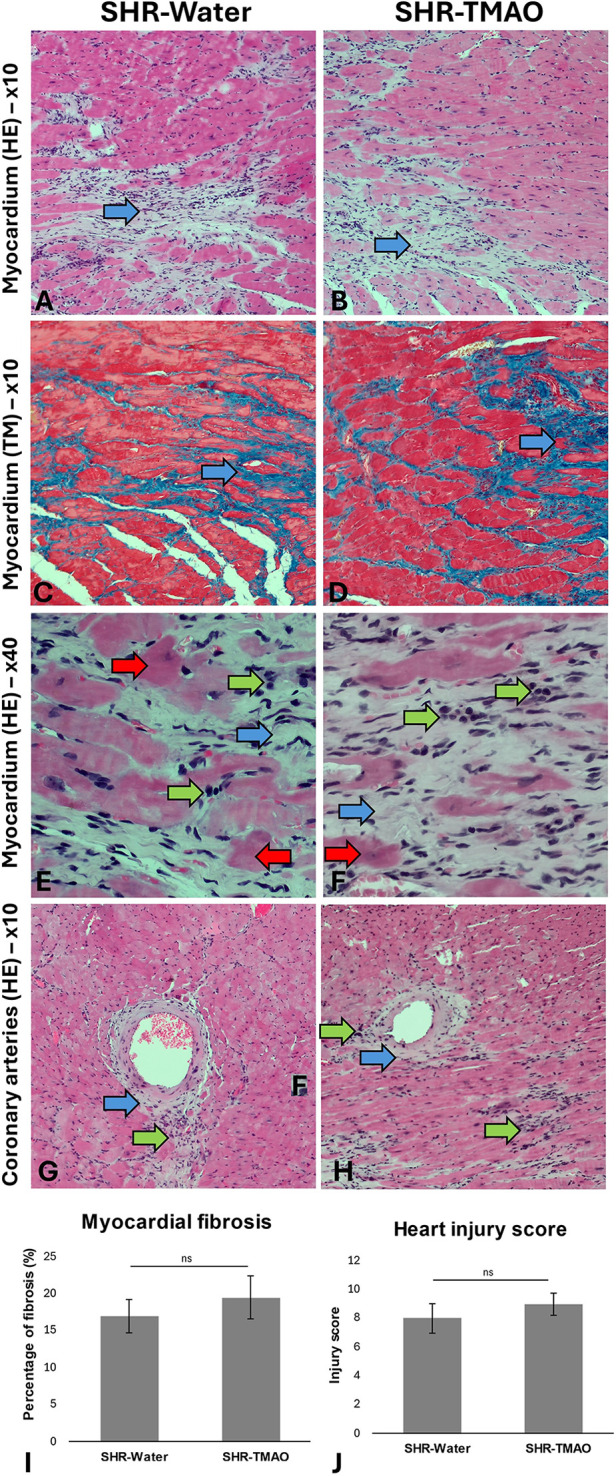
Histopathological picture of the heart in the SHR drinking either water (SHR-water) or TMAO solution (SHR-TMAO) for 80 weeks. **(A,B)**—general image of the myocardium with areas of fibrosis (blue arrow). **(C,D)**—Foci of myocardial fibrosis in Trichrom Masson staining (blue arrows). **(E,F)**—Foci of fibrosis (blue arrow) and mononuclear cell infiltration (green arrow). Degenerate, eosinophilic cardiomyocytes are also visible (red arrow). **(G,H)**—Image of coronary arteries surrounded by a band of connective tissue (blue arrow) and foci of mononuclear cell infiltration (green arrow). **(I)**—percentage of fibrosis (group means ± SE). **(J)**—heart injury score (group means ± SE).

**Figure 2:**
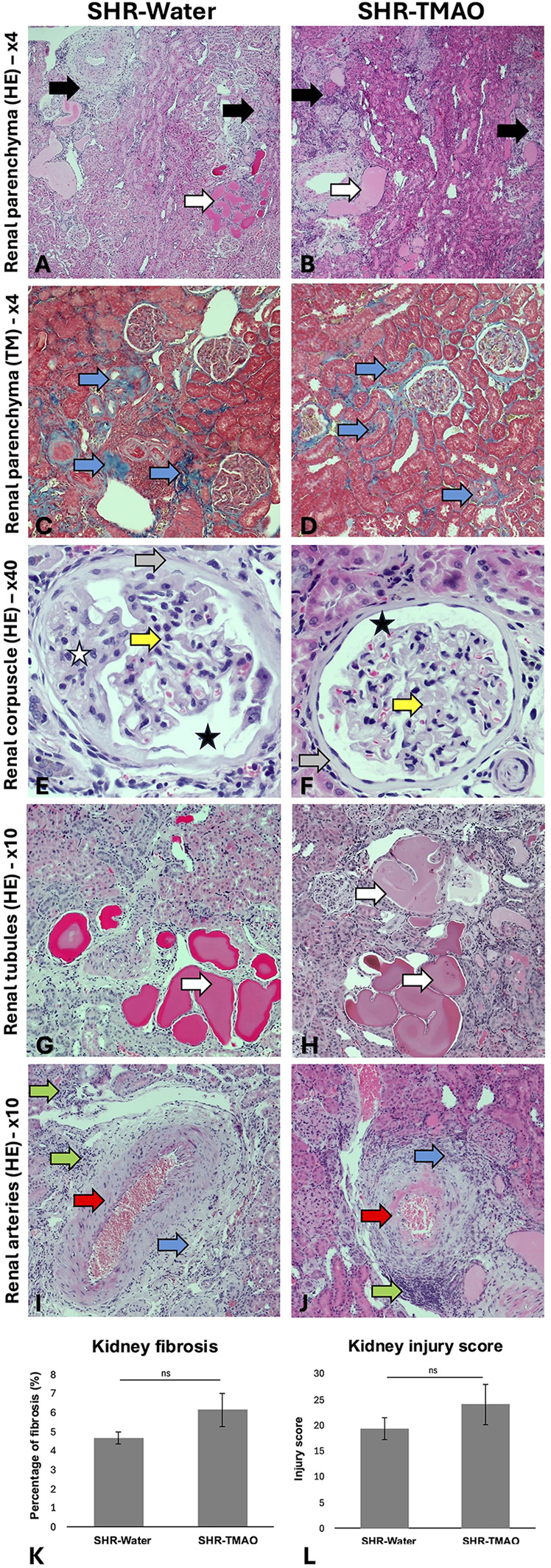
Histopathological picture of the kidneys in the SHR drinking either water (SHR-water) or TMAO solution (SHR-TMAO) for 80 weeks. **(A,B)**—general image of the renal parenchyma with visible parenchymal fibrosis (black arrow) and tubular cysts (white arrows). **(C,D)**—Fibrosis foci in the renal parenchyma in Trichrom Masson staining (blue arrow). **(E,G)**—Bowman's capsule fibrosis (gray arrow), widening of Bowman's space (black star), degenerative changes in the mesangium (white star), thickening of the basement membranes of the glomerular vessels (yellow arrow). **(G,H)**—tubular cysts in the renal parenchyma (white arrows). **(I,J)**—Image of arterial intimal hypertrophy and hyalinization (red arrow), perivascular fibrosis (blue arrow), perivascular inflammatory infiltrates (green arrow). **(K)**—percentage of fibrosis (group means ± SE). **(L)**—heart injury score (group means ± SE).

The general histological picture of heart and kidney morphology (including injury score for each evaluated parameter and summary injury score) did not differ between the groups. Morphometric analysis did not show significant differences in the degree of myocardial or kidney fibrosis between the groups (Figures 1, 2).

### 3.5 Gene expression of the tissue renin-angiotensin system

The SHR-TMAO rats had a significantly higher expression of AGT, AT1b and AT2 receptors in heart, kidney cortex, and colon in comparison to SHR-Water group.

**Figure 3:**
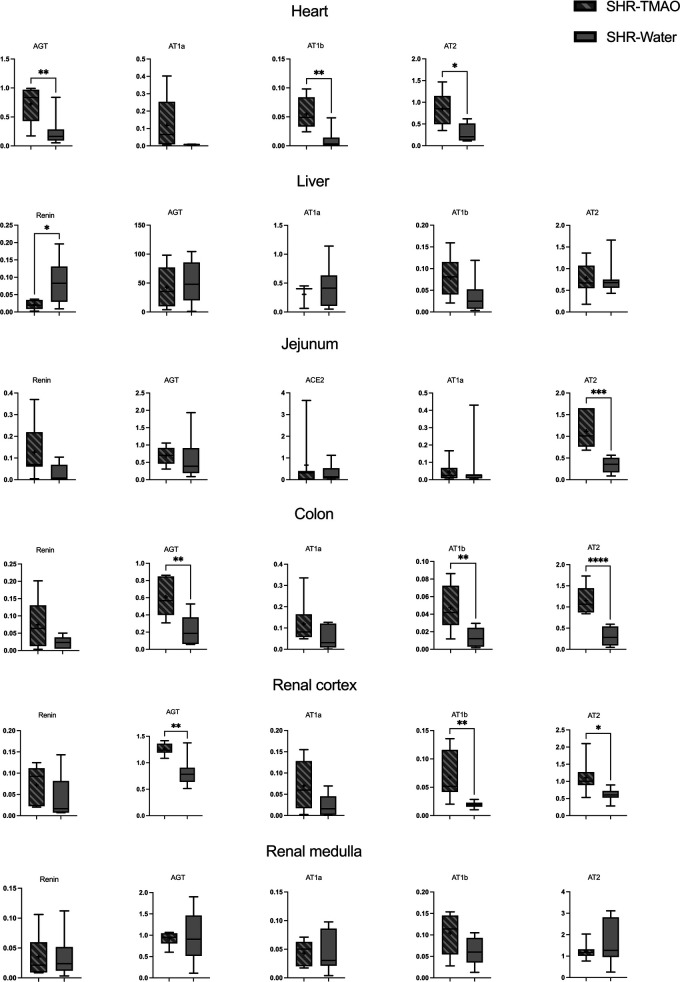
Renin-angiotensin system expression in tissues. Box plot comparing the expression proﬁles of renin, AGT (angiotensinogen), ACE2 (angiotensin converting enzyme 2), AT1a (angiotensin II receptor type 1a), AT1b (angiotensin II receptor type 1b), and AT2 (angiotensin II receptor type 2) in tissues of spontaneously hypertensive rats drinking either water (SHR-Water) or TMAO solution (SHR-TMAO) for 80 weeks. ±mean value, * indicates significant difference compared with the control group: *p < 0.05, **p < 0.01, ***p < 0.001,****p < 0.0001 by *t*-test or Mann–Whitney *U* test.

Also, SHR-TMAO group had higher expression of AT2 receptors in the jejunum, and higher renin expression in liver. There were no significant differences with respect to other RAS components in other organs (Figure 3). Cardiac renin and jejunal AT1b receptor expression were below the limit of quantification. ACE2 expressions were below the limit of quantification in most evaluated organs, except jejunum.

## 4 Discussion

In our study, we demonstrated that lifelong exposure TMAO significantly lowers arterial blood pressure and NT-proBNP levels in aged hypertensive rats. These changes, along with numerical reductions in systemic vascular resistance and left atrial size, suggest a decreased workload on the left ventricle. This effect may be facilitated by increased diuresis and natriuresis.

While cardiovascular diseases are primarily linked to aging populations, the majority of research relies on young animal models. To our knowledge, this study is the first to investigate the effects of lifelong TMAO exposure on circulatory homeostasis in rats. Also, we initiated treatment in 10-week-old rats, which begin to develop hypertension and associated cardiovascular complications (13). This positions our study as targeting prevention during the early stages of disease development, rather than focusing solely on treatment after its onset. Interestingly, rats that consumed TMAO exhibited comparable TMAO levels to those observed in our previous study using the same dose of TMAO (13). In contrast, control rats given water displayed approximately 2–3 times lower TMAO levels. While it is challenging to draw definitive conclusions in the absence of other studies on TMAO in aged rats, our current findings suggest that TMAO levels may decrease with age.

The role of TMAO in cardiovascular pathology remains a topic of ongoing debate (6). While many experimental studies have highlighted its pathological effects—such as inducing cardiac hypertrophy (7, 9), fibrosis (13), vascular aging (17), and atherosclerotic plaque formation (18)—others do not support these findings (12). Intriguingly, some studies have even suggested protective effects of TMAO precursors against heart failure (16) and atherosclerotic lesion development (14). Importantly, our laboratory previously demonstrated that a 55-week TMAO treatment, which increased plasma TMAO levels fivefold, resulted in lower diastolic blood pressure in SHR rats without causing pathological changes. Additionally, it reduced cardiac fibrosis and improved diastolic function (13). In the present study, we have shown that extending TMAO administration to 80 weeks led to a reduction in systolic and mean arterial blood pressure. Furthermore, it lowered NT-proBNP levels and left atrial size, indicating a decreased cardiac workload. This reduction in workload is further supported by trends toward decreased total peripheral resistance and heart mass, along with increased natriuresis and diuresis in TMAO-treated rats. Our findings align with reports of TMAO's diuretic and blood pressure-lowering effects in heart failure rat models (16). However, they contradict a study by Brunt et al., which found dietary TMAO increased systolic blood pressure in mice (19). These discrepancies may stem from species differences, as rats and mice vary in TMAO concentration and metabolism (20). Additionally, methodological differences in blood pressure measurement may contribute; Brunt et al. employed the tail-cuff method, whereas we used direct measurements under general anesthesia. Both methods have limitations: the tail-cuff method is prone to stress-related artifacts, while anesthesia may influence hemodynamic parameters. To resolve these inconsistencies, future studies using telemetry recordings, which avoid these limitations, are warranted.

This study did not replicate our earlier findings regarding TMAO's protective effects against cardiac fibrosis (13). Pathomorphological assessments of the heart and kidneys revealed no significant differences between the control and TMAO-treated groups. This discrepancy may be due to the differences in study designs: the current investigation involved 80-week long supplementation, with assessments conducted on significantly older rats (over 1.5 years old). It is plausible that with advancing age, the chronic effects of hypertension become so severe that some of adaptive mechanisms associated with TMAO are attenuated. Our study also aimed to evaluate the effect of lifelong TMAO exposure on survival in hypertensive rats. However, establishing a definitive impact based on our results proved challenging, as two rats from each group died before the end of the observation period. Notably, the two deaths in the SHR-Water group were attributed to apparent cardiovascular complications, specifically lung edema. In contrast, the deaths in the TMAO-treated group were unrelated to the cardiovascular system and resulted from other conditions, i.e., neoplasia and abscess formation.

While the role of TMAO remains controversial, evidence increasingly suggests that its direct precursor, TMA, has toxic effects in cardiovascular pathology. For instance, TMA has been shown to elevate blood pressure (21, 22) and induce kidney damage (22) via the ZBP1-NLRP3 inflammasome pathway (23), a mechanism previously attributed to TMAO (24). In our study, TMAO supplementation led to elevated TMA concentrations in blood and urine, potentially due to anaerobic gut bacteria converting TMAO into TMA (25) or reduction by mitochondrial amidoxime-reducing component protein (hmARC1) that can transform TMAO into TMA in tissues (26). Given the apparent toxicity of TMA, it is conceivable that it may contribute to the adverse effects of high doses of TMAO administration reported in other studies and may partially offset the beneficial effects of TMAO observed in our work.

We also examined the impact of TMAO on local tissue RAS expression. The RAS, including the local tissue RAS, is increasingly recognized as a key mediator in microbiota-host interactions (27). Its classical actions—blood pressure regulation, vasoconstriction, tissue remodeling, sodium and water retention, and pro-inflammatory effects—are primarily mediated by AT1 receptors via the ACE1-AT1R axis (28). In contrast, activation of AT2 receptors through the ACE2-MAS axis generally counteracts angiotensin II effects, promoting favorable organ changes (29). Dysregulation in the balance between these two axes is implicated in hypertension and associated organ damage (30). Previously, we observed beneficial alterations in cardiac RAS components following chronic TMAO supplementation (16). In the present study, decreased blood pressure and tendency towards increased natriuresis and diuresis in TMAO-treated rats suggested the activation of the alternative RAS axis via AT2 receptors. Its activation promotes vasodilation and natriuresis via bradykinin, nitric oxide, and cyclic GMP cascade (31). Increased AT₂R expression often emerges as a compensatory response in hypertension and ageing and is considered cardioprotective. However, we observed significantly higher expression of components from both RAS axes (AGT, AT1b, and AT2 receptors) in multiple organs, including the heart and kidney cortex. This dual upregulation might reflect a compensatory increase in systemic renin activity, triggered by the reduced blood pressure and enhanced sodium excretion observed in the TMAO-treated rats. TMAO-treated rats exhibited a higher urine output and a greater total sodium excretion, findings consistent with an osmotic diuresis generated by filtered TMAO rather than with a primary change in tubular sodium transport (16).

Our study was conducted using a single model of hypertensive rats and a single dose level of TMAO. This limitation hinders our ability to fully differentiate between age-related and hypertension-induced pathological changes and to draw conclusions on dose dependency. These constraints arose due to financial and labor costs, as well as ethical considerations regarding animal welfare in lifelong experiments.

Despite these limitations, it is important to note that our findings provide unique insights, as comparable studies are virtually nonexistent. Furthermore, our research builds on previous studies where we comprehensively assessed age- and hypertension-related changes in SHR and WKY rats (13).

## 5 Conclusions

Our study demonstrates that lifelong exposure to TMAO improves circulatory function in hypertension, as evidenced by reduced blood pressure, lower NT-proBNP levels, and decreased left atrial size—findings consistent with a reduction in cardiac workload.

However, TMAO supplementation raised blood and urinary TMA levels, potentially offsetting some benefits. Additionally, TMAO upregulated tissue RAS component expression, likely as a compensatory mechanism in response to lower blood pressure and increased natriuresis. These findings highlight the complex interplay of TMAO's effects, warranting further investigation into its long-term impacts and interaction with its precursor, TMA.

## Data Availability

The original contributions presented in the study are included in the article/Supplementary Material, further inquiries can be directed to the corresponding author.
